# Systematic Review on Global Epidemiology of Methicillin-Resistant *Staphylococcus pseudintermedius*: Inference of Population Structure from Multilocus Sequence Typing Data

**DOI:** 10.3389/fmicb.2016.01599

**Published:** 2016-10-18

**Authors:** Teresa Pires dos Santos, Peter Damborg, Arshnee Moodley, Luca Guardabassi

**Affiliations:** ^1^Department of Veterinary Disease Biology, Faculty of Health and Medical Sciences, University of CopenhagenFrederiksberg, Denmark; ^2^Department of Biomedical Sciences, Ross University School of Veterinary MedicineSt Kitts, West Indies

**Keywords:** *Staphylococcus pseudintermedius*, MLST, antimicrobial resistance, companion animals, epidemiology, systematic review, MRSP

## Abstract

**Background and rationale:** Methicillin-resistant *Staphylococcus pseudintermedius* (MRSP) is a major cause of infections in dogs, also posing a zoonotic risk to humans. This systematic review aimed to determine the global epidemiology of MRSP and provide new insights into the population structure of this important veterinary pathogen.

**Methodology:** Web of Science was searched systematically for articles reporting data on multilocus sequence typing (MLST) of *S. pseudintermedius* isolates from dogs or other animal or human patients and carriers. Data from the eligible studies were then integrated with data from the MLST database for this species. Analysis of MLST data was performed with eBURST and ClonalFrame, and the proportion of MRSP isolates resistant to selected antimicrobial drugs was determined for the most predominant clonal complexes.

**Results:** Fifty-eight studies published over the last 10 years were included in the review. MRSP represented 76% of the 1428 isolates characterized by the current MLST scheme. The population of *S. pseudintermedius* was highly diverse and included five major MRSP clonal complexes (CCs). CC71, previously described as the epidemic European clone, is now widespread worldwide. In Europe, CC258, which is more frequently susceptible to enrofloxacin and aminoglycosides, and more frequently resistant to sulphonamides/trimethoprim than CC71, is increasingly reported in various countries. CC68, previously described as the epidemic North American clone, is frequently reported in this region but also in Europe, while CC45 (associated with chloramphenicol resistance) and CC112 are prevalent in Asia. It was estimated that clonal diversification in this species is primarily driven by homologous recombination (*r/m* = 7.52).

**Conclusion:** This study provides evidence that *S. pseudintermedius* has an epidemic population structure, in which five successful MRSP lineages with specific traits regarding antimicrobial resistance, genetic diversity and geographical distribution have emerged upon a weakly clonal background through acquisition of SCC*mec* and other mobile genetic elements.

## Introduction

*Staphylococcus pseudintermedius* is a bacterial commensal of the skin and mucosae of dogs and also the most prevalent cause of canine bacterial infections (Bannoehr and Guardabassi, 2012). Although primarily adapted to the canine host, this coagulase-positive staphylococcal species may infect other pet animals, mainly cats (Kadlec et al., 2010), and humans (Van Hoovels et al., 2006). Over the last decade, multidrug-resistant strains have emerged worldwide (Black et al., 2009; Moodley et al., 2009; Kadlec et al., 2010; Perreten et al., 2010, 2013; Ruscher et al., 2010; Gómez-Sanz et al., 2011; Laarhoven et al., 2011; Osland et al., 2012; Bardiau et al., 2013; Chanchaithong et al., 2014; Couto et al., 2014; Davis et al., 2014; Savini et al., 2014; Starlander et al., 2014; Grönthal et al., 2015; Kjellman et al., 2015; Rota et al., 2015; Ishihara et al., 2016), especially clones that have acquired the Staphylococcal Chromosomal Cassette (SCC*mec*) mobilizing the methicillin resistance gene *mecA*. Infections caused by methicillin-resistant *S. pseudintermedius* (MRSP) can be difficult or even impossible to treat using veterinary licensed systemic antimicrobial agents. The proportion of MRSP amongst clinical isolates varies considerably depending on the geographic region and population studied (Norström et al., 2009; Kawakami et al., 2010; De Lucia et al., 2011; Garbacz et al., 2011; Youn et al., 2011; Feng et al., 2012; Aslantaş et al., 2013; Detwiler et al., 2013; Penna et al., 2013; Chanchaithong et al., 2014; Haenni et al., 2014; Lehner et al., 2014; Windahl et al., 2015; Ishihara et al., 2016). MRSP isolation frequencies may reach up to 67% of all clinical *S. pseudintermedius* isolates in certain countries and veterinary hospitals (Kawakami et al., 2010). Hospitalization, frequent visits to veterinary practices, and prior antimicrobial usage are recognized risk factors for canine MRSP infection and carriage (Frank et al., 2009; Rota et al., 2011; Nienhoff et al., 2011a,b; Weese et al., 2012; Windahl et al., 2012; Eckholm et al., 2013; Lehner et al., 2014; Grönthal et al., 2015).

Multilocus sequence typing (MLST) has proven to be an invaluable DNA sequence-based technique for analysis of population structure and long-term epidemiological trends at a global level (Spratt and Maiden, 1999). A first MLST scheme based on five loci was initially proposed in 2007 to provide insight into the overall population genetic structure of the *Staphylococcus intermedius* Group (SIG), which includes *S. intermedius* and *S. delphini* in addition to *S. pseudintermedius* (Bannoehr et al., 2007). Using this scheme two major epidemic MRSP clones were detected, ST68 in North America and ST71 in Europe (Perreten et al., 2010). The first species-specific MLST scheme was launched in 2013 (Solyman et al., 2013), and its publicly available database (http://pubmlst.org/spseudintermedius/) contains records of 503 sequence types (STs) at present. Since 2007, numerous scientific papers have reported MLST data on MRSP carriage and infection in a variety of countries and continents, but to date these studies have not been reviewed systematically.

The aim of this systematic review was to provide an updated overview of the global epidemiology and evolution of MRSP, with a focus on geographical distribution of reported frequency, SCC*mec* content and antimicrobial resistance of the main clonal lineages, as defined by MLST. The results of the review were also used to infer the population structure of the species, including methicillin-susceptible *S. pseudintermedius* (MSSP), as well as to discuss the evolutionary mechanisms that could be implicated in the diversification of MRSP clones.

## Methodology

### Search strategy and study selection criteria

**Figure 1:**
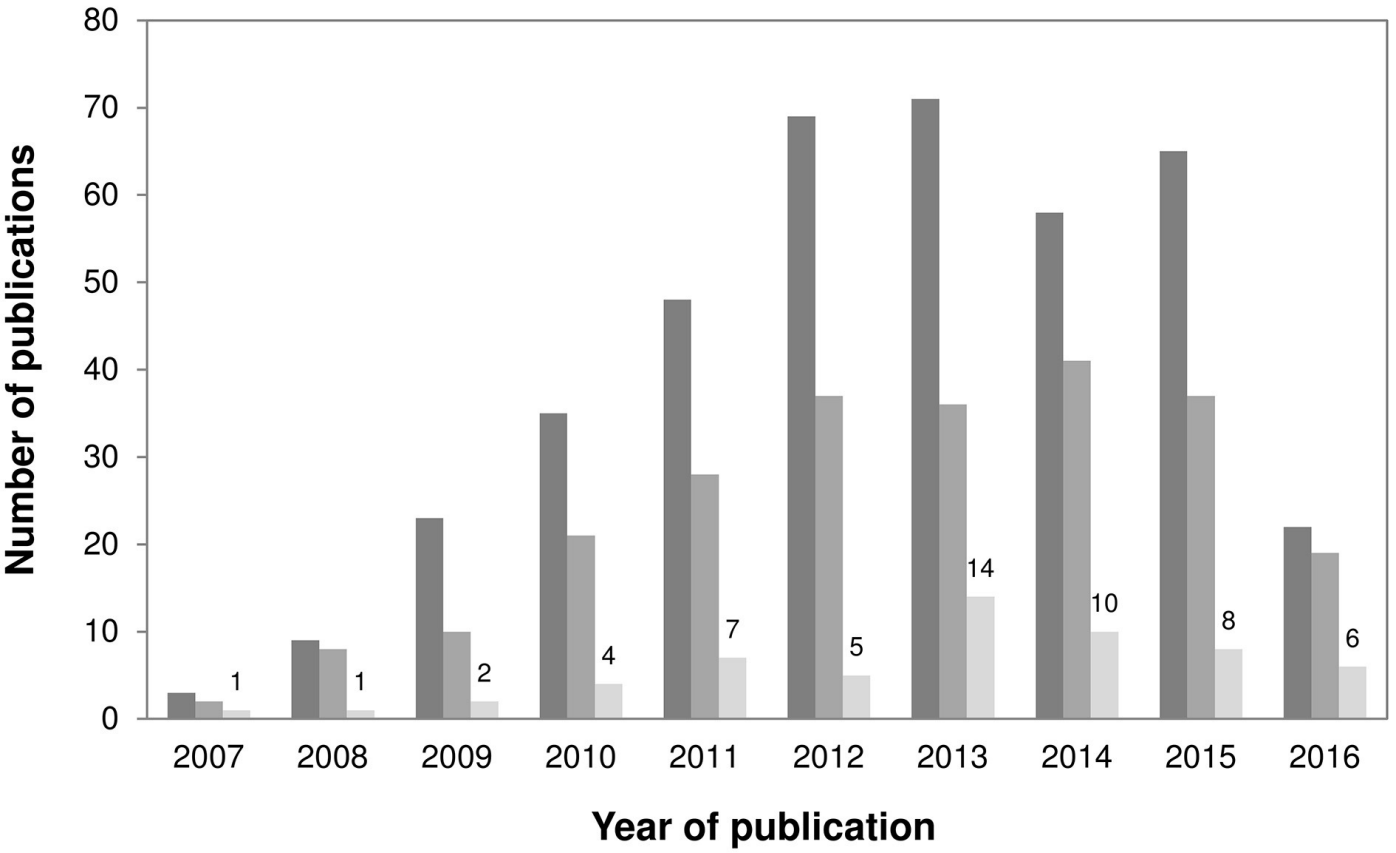
**Bar chart showing (i) the annual number of published articles retrieved when using the search word “*Staphylococcus pseudintermedius”* (dark gray); (ii) the annual number of published articles retrieved involving MRSP (intermediate gray); and (iii) the annual number of published articles retrieved employing MLST (light gray), since 2007–May 2016**. A total of 58 studies reporting MLST data on MRSP and MSSP were included in this review.

We searched the Web of Science database for articles published between 28th September 2007 and 31st May 2016 using the search term “*Staphylococcus pseudintermedius*.” This resulted in the retrieval of 403 articles, of which 239 reported data on MRSP. Studies were then selected if they reported MLST data on MRSP and/or MSSP isolates from screening and/or clinical samples, including data obtained by the 5-allele (Bannoehr et al., 2007; MLST-5) or the 7-allele (Solyman et al., 2013; MLST-7) scheme. Studies that exclusively used *dru* typing (*n* = 5) to characterize *S. pseudintermedius* strains were not included. These steps yielded 58 studies (Figure 1), including 49 studies on MRSP (with or without MSSP; Bannoehr et al., 2007; Descloux et al., 2008; Black et al., 2009, 2011; Moodley et al., 2009, 2013; Kadlec et al., 2010, 2015, 2016; Perreten et al., 2010, 2013; Ruscher et al., 2010; Stegmann et al., 2010; Boost et al., 2011; Gómez-Sanz et al., 2011, 2013a,b, 2014; Laarhoven et al., 2011; Paul et al., 2011; DiCicco et al., 2012, 2014; Feng et al., 2012; Osland et al., 2012; Walther et al., 2012; Wang et al., 2012; Bardiau et al., 2013; Haenni et al., 2013, 2014; Pilla et al., 2013; Quitoco et al., 2013; Solyman et al., 2013; Youn et al., 2013; Chanchaithong et al., 2014, 2015; Couto et al., 2014, 2016; Davis et al., 2014; Grönthal et al., 2014, 2015; Savini et al., 2014; Starlander et al., 2014; Börjesson et al., 2015; Kjellman et al., 2015; McCarthy et al., 2015; Rota et al., 2015; Damborg et al., 2016; Duim et al., 2016; Ishihara et al., 2016) and 9 studies on MSSP only (Ben Zakour et al., 2011; Tse et al., 2011; Gharsa et al., 2013, 2015; Matanovic et al., 2013; Paul et al., 2013; Gómez-Sanz et al., 2013c; Youn et al., 2014; Lozano et al., 2015).

### Data extraction and review approach

A database was created to record location, date of isolation, source, host, resistance phenotype, resistance genotype, and ST of each isolate. Susceptibility data were based on the interpretation criteria used in the original articles, and isolates reported as “intermediate” were considered “susceptible.” Only one isolate per animal was included, unless multiple STs were reported in the same animal. When two isolates from the same animal belonged to the same clone but differed in antimicrobial susceptibility patterns, only the isolate displaying resistance to most drug classes was considered. The information collected in the scientific literature was integrated with that available in the *S. pseudintermedius* MLST database (26 June 2015), incorporated into an Excel file and summarized in Table S1. Stringent cross-checking of strain information in the database and in the papers was performed to ensure that strains were only accounted for once. Statistical comparisons of the relative prevalences of resistance were based on the chi-square test of independence or, for small numbers, on the Fisher's exact.

### Bioinformatic analysis

eBURST V3 (Feil et al., 2004) (http://eburst.mlst.net) was used to infer the patterns of evolutionary descent from MLST allelic data. The program settings were adjusted to define clonal complexes (CCs) as groups of sequence types (STs) sharing at least six identical alleles, with the primary founder being the ST having the most number of single locus variants (SLVs). Subgroup founders were defined according to the default settings of eBURST, i.e., as STs with at least three SLVs that diversified from a primary founder. A bootstrapping resampling procedure (1000 samples) was used to assess the statistical confidence in the primary founders.

Validation of ST memberships in various CCs was done with phylogenetic analysis based on the nucleotide sequences of the *S. pseudintermedius* MLST genes (*ack, cpn60, fdh, pta, purA, sar*, and *tuf*; Solyman et al., 2013). A total of 144 STs representing major eBURST group/subgroup founders (with more than three SLV links) and singletons with no double locus variant (DLV) links to eBURST-assigned CCs (Willems et al., 2011) were used in the analysis. Two independent ClonalFrame (Didelot and Falush, 2007) runs consisting of 2,000,000 MCMC iterations were performed, the first half of which was discarded as burn-in. The Gelman-Rubin statistic was calculated to assess convergence of the two runs (< 1.1) (Vos and Didelot, 2009), and the genealogy was built as a 50% majority-rule consensus tree. The Bayesian posterior probability of a given cluster (i.e., the proportion of trees supporting a given branch) was used to evaluate the confidence level in the assigned clusters; clusters with confidence levels >0.90 were considered well supported.

The rates at which recombination (rho, ρ) and mutation (theta, θ) occur were extracted from one ClonalFrame output file to assess how frequent recombination events occur relative to mutation events (ρ*/*θ). The ratio of probabilities that a given site is altered through recombination and mutation (*r/m*) was also estimated to determine how important the effect of recombination has been to clonal diversification relative to mutation. The program MultiLocus (Agapow and Burt, 2001) (version 1.2, Imperial College, available at http://www.bio.ic.ac.uk/evolve/software/multilocus) was used to test for significant linkage disequilibrium among the MLST loci (1000 randomizations were used for all *P*-value estimates).

Genotypic (ST) diversity (*G*), i.e., the probability that two randomly picked isolates belong to a different ST; genetic (allelic) diversity of a locus (*h*), i.e., the probability that two randomly picked STs have a different allele at a locus; and mean allelic diversity over the seven loci (*H*) were calculated with the software Arlequin V3.1 (Excoffier et al., 2005). Analysis of molecular variance (AMOVA) as a weighted average over loci was carried out to determine the degree of genetic variation between subpopulations (MRSP and MSSP). Descriptive statistics for MLST data including the number of variable sites/codons, nucleotide diversity and estimates of *d*_S_ and *d*_N_ (Tamura-Nei model) were performed using MEGA V7.0.14 (Kumar et al., 2016).

## Results

### Sources of *S. pseudintermedius* isolates

MRSP represented 1087 (76.1%) of all 1428 *S. pseudintermedius* isolates that were characterized by MLST-7, and 558 (76.1%) of all 733 *S. pseudintermedius* isolates that were characterized according to MLST-5. Approximately 14.3% of MRSP isolates (*n* = 155) were characterized according to both schemes. Clinical isolates accounted for 81.8% of the isolates analyzed by MLST-7 for which an isolation source could be identified (*n* = 892). These were mainly isolated from skin samples associated to pyoderma, surgical site- and wound infections (64.1%). MRSP accounted for 80.5% of all 892 isolates, for 86.2% of clinical isolates and for 54.9% of carriage isolates. Most (90.5%) isolates of known source originated from dogs (*n* = 804, of which 83.3% were MRSP), while the remaining isolates were recovered from humans (*n* = 36, 52.8% of which were MRSP), cats (*n* = 26, 73.1% of which were MRSP) and horses (*n* = 3, of which one was MRSP). Among the 36 isolates from human origin, 22 were from clinical specimens (31.8% of which were MRSP) while 14 were from screening samples (85.7% of which were MRSP) (Table S1).

### Nucleotide sequence variation at each MLST locus

A high genotypic diversity (*G* = 0.8720; *SD* = 0.0082) was detected among the 1428 isolates characterized by MLST-7. Among the 503 existing STs, 431 were reported only once, while 72 were reported multiple times. Sequence comparisons amongst all STs revealed an average of 18.4 variable nucleotide sites (range = 7–29) and 3.4 variable codons (range = 1–8) per locus (Table 1). The average number of alleles at the seven housekeeping genes was 25.4, ranging from 8 alleles for *tuf* to 44 for *cpn60* and *purA*. The average single-locus genetic diversity (*H*) was high for the total population (*H* = 0.710; *SD* = 0.060). The genes *cpn60, fdh*, and *purA* showed higher nucleotide diversity (π = 0.011–0.058) than *ack, pta, sar*, and *tuf* (π = 0.005–0.008). There was a low percentage of nonsynonymous (amino-acid changing) substitutions for all seven genes (*d*_S_/*d*_N_ = 3.557–35.090) (Table 1), indicating that natural selection acting on housekeeping genes is negative (*d*_S_/*d*_N_ > 1, i.e., purifying selection), as it is expected for genes encoding essential cellular functions.

**Table 1:** **Nucleotide variation within seven housekeeping genes analyzed by MLST**.

**MLST locus**	**Sequence length (bp)**	**No. of alleles**	**No. of variable sites (%)**	**No. of variable codons (%)**	**Single-locus heterozygosity (*h*)**	**Nucleotide diversity (π)**	***d_S_*/*d_N_* ratio**
*ack*	564	21	13 (2.3)	2/174 (1.1)	0.807	0.005	18.270
*cpn60*	431	44	25 (5.8)	2/91 (2.2)	0.895	0.011	35.090
*fdh*	259	13	12 (4.6)	3/71 (4.2)	0.707	0.013	3.557
*pta*	492	29	29 (5.9)	5/84 (6.0)	0.584	0.008	4.880
*purA*	405	44	25 (6.2)	8/99 (8.1)	0.897	0.058	6.598
*sar*	376	19	18 (4.8)	3/88 (3.4)	0.566	0.008	7.753
*tuf*	417	8	7 (1.7)	1/116 (0.9)	0.514	0.005	12.321
All 502 STs^a^	2944	25.4	129 (4.4)	24/723 (3.3)	0.710	0.004	25.451

a *One ST (ST392) differed markedly from the others in the pta gene and was therefore not included in the analyses of diversity*.

### Distribution of MRSP and MSSP isolates

The obtained data included 335 MSSP isolates that belonged to 328 STs, and 1087 MRSP isolates that belonged to 182 STs. Genotypic diversity was higher among MSSP (*G* = 0.9999; *SD* = 0.0002) than among MRSP (*G* = 0.7791; *SD* = 0.0126). Sequence type 71 was the most predominant ST amongst MRSP (44.9%), followed by ST45 (11.0%), ST258 (6.9%), ST261 (3.5%), ST112 (1.9%), ST265 (1.7%), ST68 (1.5%), ST169, and ST181 (1.4%), which were reported ≥15 times and represented 74.2% of all 1087 MRSP isolates. Only seven STs were common to both MRSP and MSSP isolates (ST17, ST29, ST56, ST123, ST125, ST155, and ST170). AMOVA showed a small but significant genetic variation (2.31%) between MRSP and MSSP subpopulations (*P* < 0.05), indicating that they are slightly genetically distinct.

### Identification of main clonal lineages

**Figure 2:**
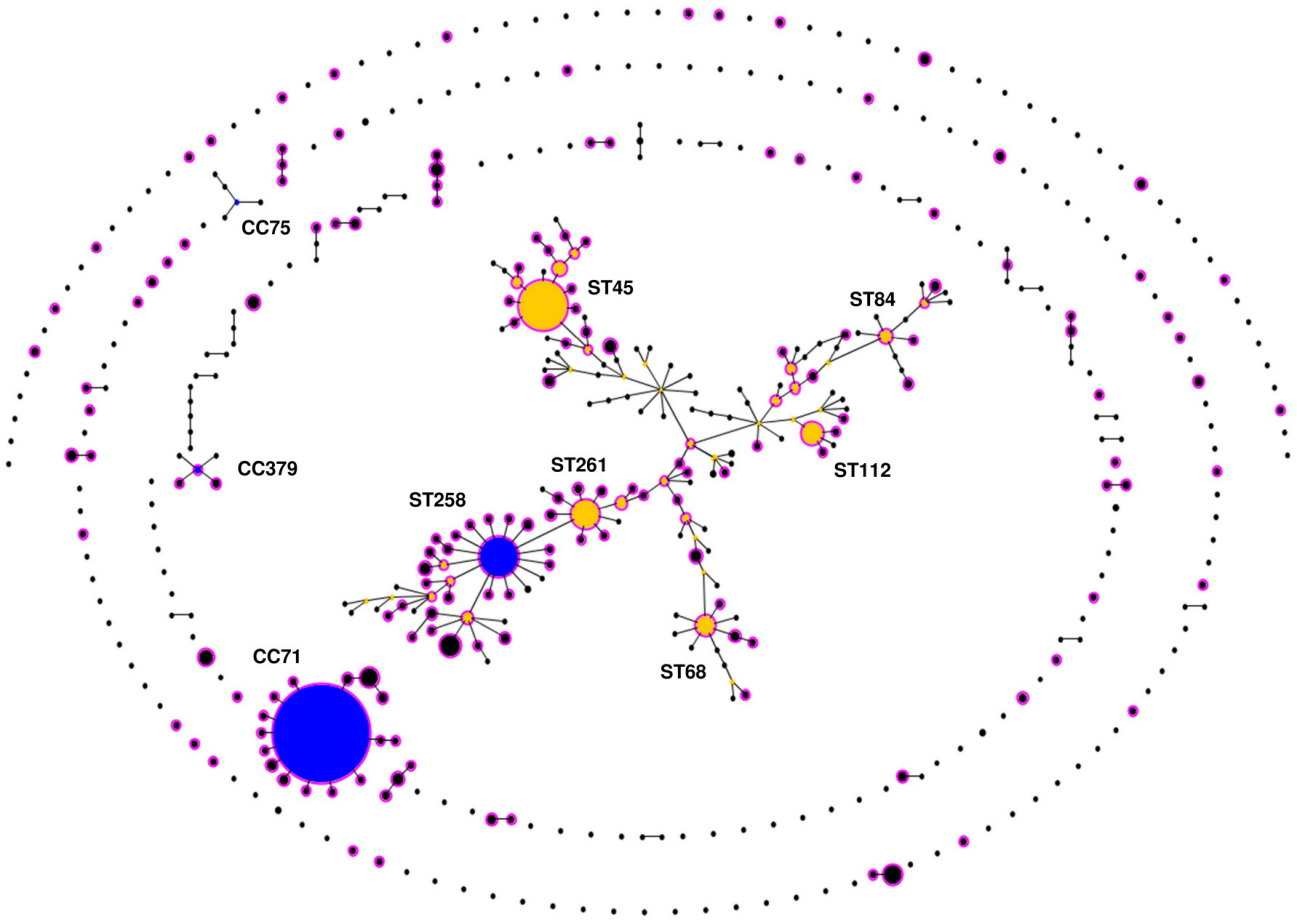
**eBURST population snapshot of *Staphylococcus pseudintermedius***. Clusters of linked isolates correspond to clonal complexes (CCs). Primary founders (blue) are positioned centrally in the clusters, and subgroup founders are shown in yellow. The diameter of the circles is directly proportional to the total number of isolates reported in the MLST database (http://pubmlst.org/spseudintermedius/) and in reviewed scientific papers given in Table S1. Names of various important STs associated with methicillin resistance are indicated within a large, “straggly” CC, CC258. Methicillin resistant *S. pseudintermedius* (MRSP) lineages are highlighted in pink.

The 503 STs of *S. pseudintermedius* were assigned by eBURST into two major (CC71 and CC258) and two minor CCs (CC379 and CC75), 30 groups of STs with no predicted founder (or a founder that was not well supported; bootstrapping < 50%), and 231 singletons (Figure 2). While CC258 and CC379 comprised both MRSP and MSSP isolates, CC71 only comprised MRSP and CC75 only comprised MSSP. CC71 was largely dominated by ST71 and included 15 additional STs (12 SLVs, 2 DLVs, and 1 TLV). In contrast, CC258 formed a large straggly cluster accounting for 34.4% (*n* = 173) of all STs and 46.7% (*n* = 85) of the STs associated with MRSP. The remaining MRSP isolates were widely distributed throughout the population of *S. pseudintermedius* in a wide variety of different lineages (Figure 2, in pink).

### Phylogenetic analysis

**Figure 3:**
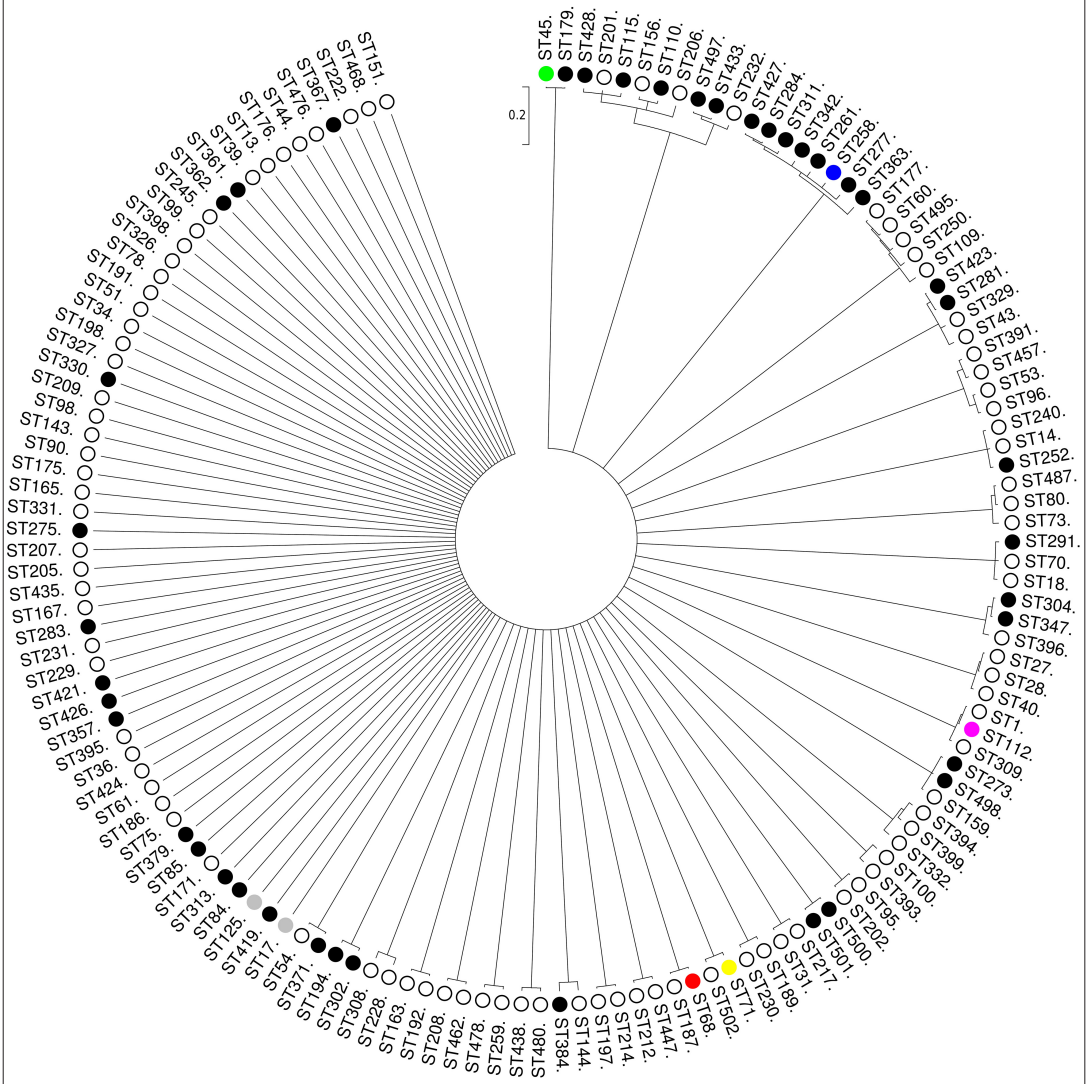
**Unrooted consensus tree displaying the relationship between the 144 STs of the *S. pseudintermedius* sample (96 MSSP, 46 MRSP, and 2 MSSP/MRSP) at seven concatenated loci (2944 nucleotides in total)**. The 144 STs represent major eBURST group/subgroup founders (with more than three SLV links) and singletons with no double locus variant (DLV) links to eBURST-assigned CCs. The phylogenetic tree was based on all trees sampled after 2,000,000 iterations, from which half were discarded as burn-in. The dendogram was constructed from the combination of two ClonalFrame runs with a cut-off value of 0.5 as a majority rule consensus. Scale is in coalescent units. There were no well-supported nodes (i.e., with a Bayesian posterior probability > 0.90) for clusters with > 3 STs). The main MRSP clones are shown in different colors. MRSP, black; MSSP, white; both MRSP and MSSP, gray.

Phylogenetic analysis of the concatenated sequences of the seven MLST loci from 144 STs using ClonalFrame showed that MRSP were associated to a variety of different STs and often clustered with MSSP (Figure 3). However, the major group/subgroup founders (e.g., ST45, ST68, ST112, and ST258) that were linked together using eBURST (Figure 2) appeared as distinct phylogenetic lineages that do not share a recent common ancestor (Figure 3). Therefore, in our subsequent analyses, we used arbitrary CC designations (e.g., CC45, CC68, CC112, and CC258) that include the founder STs confirmed by ClonalFrame to represent phylogenetically distinct lineages, and their respective single-, double-, and triple locus variants (SLV, DLV, and TLV), as defined by eBURST.

The clustering performed by eBURST and a phylogenetic tree built with all 503 STs (Figure S1) were in general agreement for CC71, i.e., 10 of the 16 STs from this CC clustered with high confidence (1.00) with ClonalFrame. Likewise, eight of the 15 STs from CC68 were clustered with high confidence (0.99) in the ClonalFrame phylogenetic tree, together with three eBURST singletons that were DLVs of STs belonging to CC68 (Figure S1). In contrast to CC71 and CC68 though, CC258 lineages appeared as part of a bundle of STs within a star-shaped genealogy (503 STs) or, for the analyzed 144 STs, in a node that was not well supported (0.67) (Figure 3). In the case of CC258, only eBURST could provide a hypothesis about the way this CC has emerged and diversified.

ClonalFrame analysis revealed that recombination occurred more frequently (ρ/θ = 3.68) and had a more important contribution to clonal diversification of *S. pseudintermedius* than mutation (*r/m* = 7.52). The standardized value of the index of association between the alleles, although much lower than 1, was still significantly different from 0 (*rbar*_d_ = −0.1218, *P* < 0.001), indicating that there is weak but significant linkage disequilibrium between the MLST loci, and ultimately that the *S. pseudintermedius* population is weakly clonal. Similar results were obtained when analyzing separately the 182 STs found amongst MRSP (*rbar*_d_ = 0.0312, *P* < 0.001), whereas there was no significant linkage disequilibrium between the MLST loci of the 328 STs detected amongst MSSP (*rbar*_d_ = −0.1521, *P* = 0.065) nor for the STs from highly supported clusters (e.g., CC71, CC68; Figure S1), a result that reinforces the recombinogenic nature of *S. pseudintermedius*.

### Geographical dissemination of the main MRSP lineages

**Figure 4:**
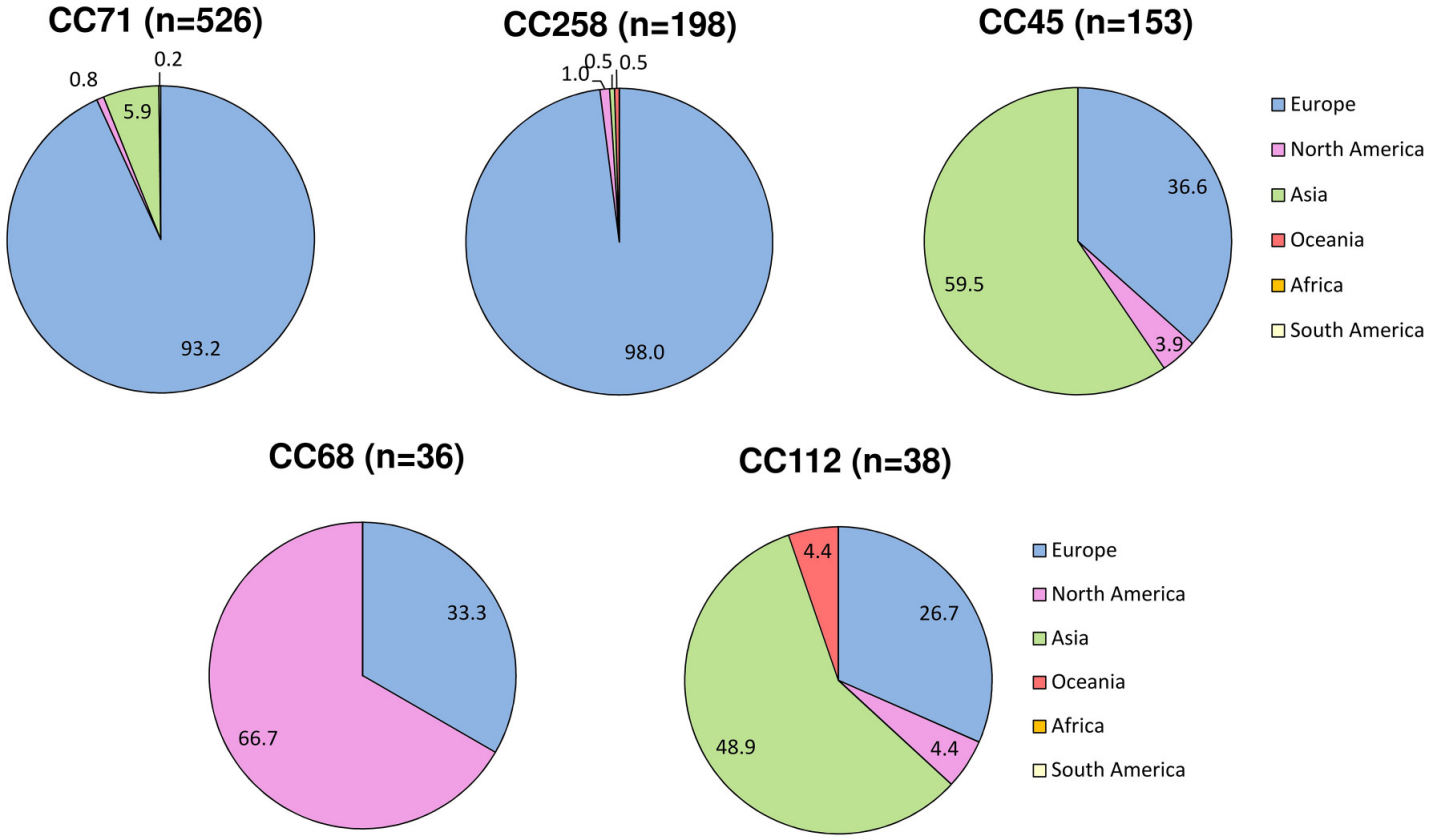
**Geographic variation in the reported frequency of major MRSP clonal complexes based on data from the MLST database (http://pubmlst.org/spseudintermedius/) and references given in Table S1**. The numbers represent the percentage (%) of MRSP isolates from each lineage that was reported in each continent.

Overall, 24 countries were represented in our dataset (Table S1). Twenty-five STs, 20 STs of which associated with MRSP, were recovered from multiple countries and continents. The majority of MRSP isolates were recovered from Europe (70.6%), followed by Asia (22.4%), North America (6.2%), Oceania (0.6%), and South America (0.2%). Among the 182 MRSP-associated STs, 16 were recovered from more than 1 continent (ST17, ST29, ST45, ST68, ST71, ST85, ST112, ST121, ST123, ST125, ST155, ST169, ST179, ST180, ST282, and ST261), and 34 were reported in more than 1 country. The most widely disseminated clone was ST71, which was isolated in 14 different countries across 3 continents (Europe, North and South America, and Asia). The other widespread MRSP lineages were distributed across two continents and included ST45 (7 countries); ST121 and ST261 (4 countries); ST68, ST169, ST265, and ST277 (3 countries); and ST17, ST29, ST85, ST112, ST123, ST125, ST155, ST179, ST180, and ST188 (2 countries). ST41, ST118, ST181, ST196, ST258, ST265, ST277, ST298, ST301, ST307, ST312, ST339, ST342, and ST414 were reported in multiple countries but one continent only. Certain MRSP clonal lineages were frequently reported in particular continents, namely CC71 and CC258 in Europe (93 and 98%, respectively); CC68 in North America (66.7%); and CC45 and CC112 in Asia (59.5 and 48.9%, respectively; Figure 4).

### Distribution of SCC*mec* among main MRSP clonal lineages

The distribution of SCC*mec* types (when reported) varied significantly (*P* < 0.0001) across distinct MRSP clonal groups (Table S1). A total of five SCC*mec* types were reported: (i) SCC*mec* type II-III was significantly associated with CC71; (ii) type ψSCC*mec*_57395_ was significantly associated with CC45; (iii) SCC*mec*_AI16_-SCC*czr*_AI16_-CI was significantly associated with CC112 isolates; and (iv) SCC*mec* type IV was significantly associated with CC258. In contrast, SCC*mec* type V was present in CC68, CC45, and CC379, as well as in 21 STs unassigned to any CC, indicating both vertical and horizontal transfer of SCC*mec* into *S. pseudintermedius* of different genetic backgrounds. Based on the patterns of evolutionary descent defined by eBURST and available SCC*mec* typing data, we estimated that *S. pseudintermedius* has acquired SCC*mec* at least 48 times.

### Antimicrobial resistance patterns of main MRSP clonal lineages

**Figure 5:**
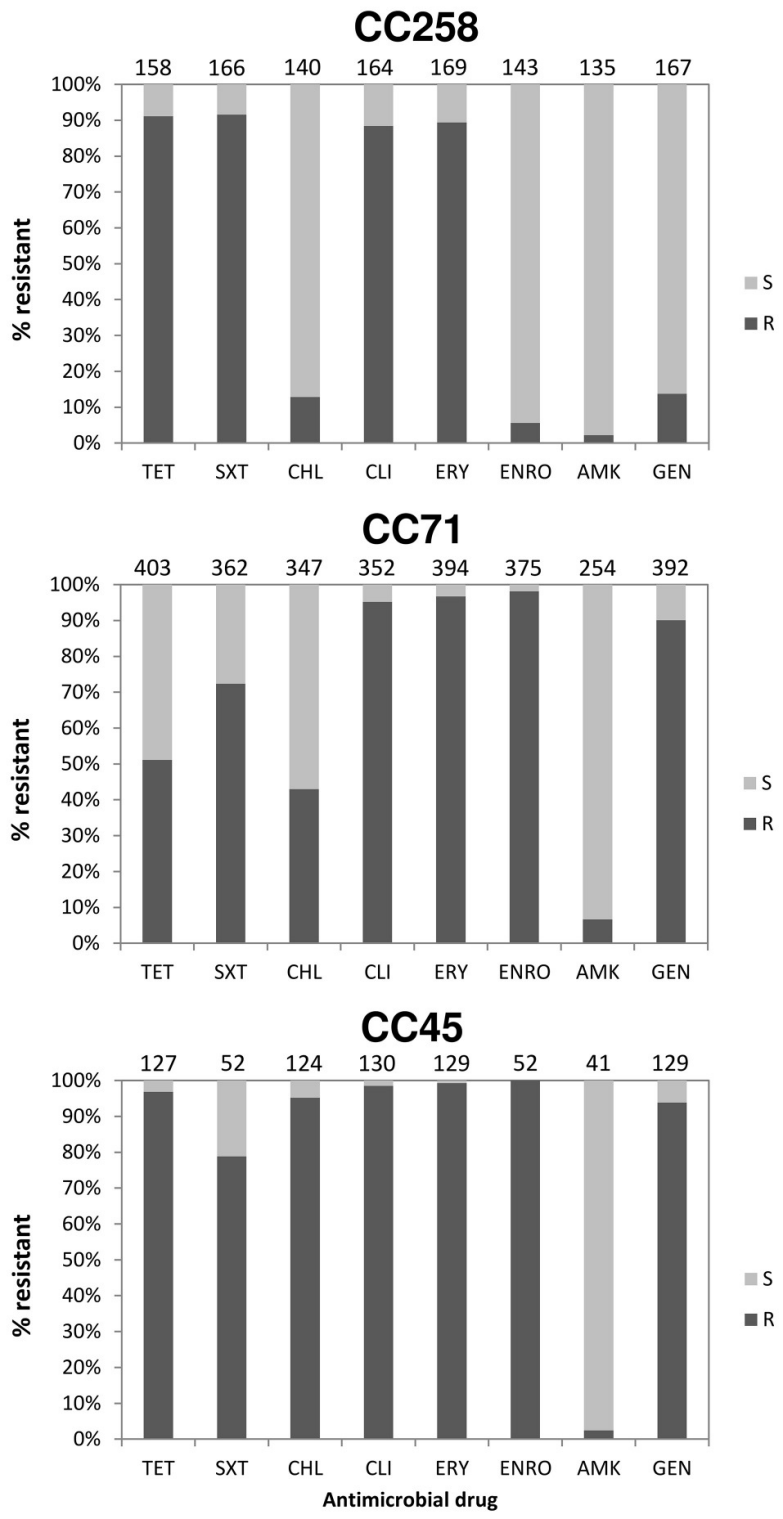
**Proportion (%) of MRSP isolates resistant to selected antimicrobial drugs within three predominant clonal complexes (characterized by MLST-7)**. Antimicrobial susceptibility patterns were extracted from references given in Table S1. S, Susceptible; R, Resistance; TET, Tetracycline; SXT, Trimethoprim/Sulfamethoxazole; CHL, Chloramphenicol; CLI, Clindamycin; ERY, Erythromycin; ENRO, Enrofloxacin; AMK, Amikacin; GEN, Gentamicin.

The overall prevalence rates of resistance amongst MRSP isolates to selected non-β-lactam antimicrobials were: erythromycin, 93.5% (762/815); clindamycin, 92.7% (695/750); tetracycline, 70.7% (573/810); trimethoprim/sulfamethoxazole, 76.6% (490/640); enrofloxacin, 73.8% (454/615); gentamicin, 71.3% (574/805); chloramphenicol, 43.1% (304/706); and amikacin, 5.2% (21/404). The prevalence of antimicrobial resistance in the three most prevalent MRSP clonal groups (CC71, CC45, and CC258) are shown in Figure 5. CC258 showed significantly lower prevalence of resistance to gentamicin and enrofloxacin (13.8 and 5.6%, respectively) as compared to CC71 and CC45 (90.1–93.8 and 98.1–100.0%, respectively) (*P* < 0.0001), but significantly higher prevalence of resistance to trimethoprim/sulfamethoxazole (91.6%) as compared to CC71 (72.3%, *P* < 0.0001) and CC45 (78.8%, *P* = 0.012). Chloramphenicol resistance was significantly more prevalent in CC45 (95.2%) compared to CC71 (42.9%) and CC258 (12.9%) (*P* < 0.0001), and the prevalence of tetracycline resistance was significantly lower for CC71 isolates (51.1%) compared to CC258 and CC45 (91.1 and 96.9%, respectively) (*P* < 0.0001). MRSP CC71 was also significantly associated with carriage of the tetracycline resistance gene *tet*(K), either alone or together with *tet*(M) (*P* < 0.0001), while non-CC71 lineages were significantly associated with carriage of *tet*(M) alone (*P* < 0.0001).

## Discussion

This is the first study providing insights into the global epidemiology of MRSP and population structure of *S. pseudintermedius* based on the recently established MLST-7 scheme for this species. Despite the extensive genetic diversity observed at the species level, 25 STs, 20 of which associated with MRSP, were reported in geographically-distant countries, indicating global spread of multiple high-risk MRSP clones.

Occurrence of the same clones over broad geographical areas is usually taken as evidence of clonal population structure. However, high levels of recombination (*r/m* = 7.52) and the index of association between the MLST loci (*rbar*_d_ = −0.1218, *P* < 0.001) revealed by our population genetics analyses show that this species is only weakly clonal. These results are consistent with an eBURST population snapshot where >25% of the STs belong to a large straggly CC (CC258), a pattern that points to potentially inaccurate links and that is often observed in populations with high recombination to mutation ratios (e.g., *Burkholderia pseudomallei, Staphylococcus epidermidis*, and *Enterococcus faecium*; Vesaratchavest et al., 2006; Miragaia et al., 2007; Turner et al., 2007; Willems et al., 2011). This clustering pattern was also recognized by Perreten et al. (2010) when using eBURST on *S. pseudintermedius* MLST-5 data alone. The term “epidemic clonal,” or simply “epidemic,” has been used to describe population structures of this kind, where particularly successful lineages emerge and diversify rapidly within a weakly clonal- or non-clonal population (e.g., *Neisseria meningitidis*; Maynard Smith et al., 2000; Vos and Didelot, 2009). This is in contrast to highly clonal species such as *Staphylococcus aureus*, which shows a very low recombination to mutation ratio (*r/m* = 0.01; Vos and Didelot, 2009) and discrete clonal lineages diversifying radially from phylogenetically well-supported founders (Feil et al., 2003). Indeed, the ClonalFrame-based phylogenetic analysis confirmed that inter-lineage recombination has likely caused incorrect links within the larger straggly group identified by eBURST. Furthermore, the genealogy shown for *S. pseudintermedius* is star-shaped (Figure 3), i.e., deep-level phylogenetic relationships could not be inferred, which often occurs when rates of recombination are high (Spratt and Maiden, 1999).

Both eBURST and ClonalFrame analyses showed that MRSP is found in association with numerous unrelated clones throughout the *S. pseudintermedius* population. These results are in agreement with recent whole genome sequencing data showing that methicillin resistance has likely evolved in genetically distinct *S. pseudintermedius* lineages through multiple independent SCC*mec* acquisitions (McCarthy et al., 2015). We estimated that *S. pseudintermedius* has acquired SCC*mec* at least 48 times, a value similar to that reported for *S. epidermidis* (56 times; Miragaia et al., 2007), and higher than that reported for *S. aureus* (at least 20 times) (Robinson and Enright, 2003). The reason for the higher number of SCC*mec* acquisitions in *S. pseudintermedius* in comparison to *S. aureus* might be due to an increased capacity for horizontal gene transfer, as was hypothesized by Miragaia et al. for *S. epidermidis* (Miragaia et al., 2007). However, the lack of complete SCC*mec* typing information for MRSP and the fact that at least 23 SCC*mec* acquisitions have occurred for one MRSA clone alone (Nübel et al., 2008) indicate that these eBURST-based estimates are likely to be underestimated.

Despite the lack of a recent common ancestor, MRSP lineages are, collectively, genetically distinct from MSSP, as shown by a small but significant genetic variation between the two subpopulations (2.31%, AMOVA: *P* < 0.05). This is also illustrated by the fact that only seven STs (ST17, ST29, ST56, ST123, ST125, ST155, and ST170) were common to both MRSP and MSSP, further strengthening that MRSP form a polyclonal *S. pseudintermedius* subpopulation composed of evolutionarily distinct lineages. The clustering performed by eBURST and ClonalFrame agreed to some extent for some CCs (e.g., CC71, CC68), supporting the clonal evolutionary model determined by eBURST. Our findings regarding CC258 further support previous evidence that the model of bacterial evolution implemented in eBURST is one of the most suitable for inferring patterns of evolutionary descent from MLST data over short periods of time when rates of recombination and clonal diversification are high (Feil et al., 2004).

This study also provides an updated overview of the geographic distribution of reported frequencies of five major CCs that have acquired methicillin resistance. MRSP CC71, which has been originally described as the epidemic European clone (Perreten et al., 2010), has spread worldwide, even though the vast majority of the isolates belonging to this CC have been reported in European countries (approximately 93%). Our observation that ~98% of CC258 isolates have been recently reported in Europe (2012–2016) confirms that CC258 may be partly replacing CC71, at least in certain European countries, as recently shown by a longitudinal study in the Netherlands (Duim et al., 2016). Geographical associations were however less marked for the other three main CCs. Approximately 33% of the isolates belonging to CC68, previously denominated as the epidemic North American clone (Perreten et al., 2010), originated from Europe. Approximately 67% of the CC45 and 49% of CC112 isolates originated from Asian countries, with the remaining isolates from the latter two CCs being isolated in Europe and to a lesser extent in North America and, for CC112, also in Oceania (Figure 4). Too few isolates originated from Africa and South America to infer clonal distribution in these continents. The different distribution of reported frequencies of MRSP clones across different countries and continents is likely to reflect various factors, including geographic distribution of *S. pseudintermedius* diversity and national patterns of antimicrobial usage.

In addition, significant differences were observed in the prevalence of antimicrobial resistance across distinct MRSP clonal groups. For instance, MRSP CC258 isolates display a very distinct resistance profile compared to CC71 and CC45, i.e., enrofloxacin and gentamicin resistance was infrequent in CC258 (6 and 14%), whereas resistance to trimethoprim/sulfamethoxazole was more frequent in CC258 (92%) than in the other two CCs (73–79%); tetracycline resistance was less frequent in CC71 isolates (51%) compared to those of CC258 and CC45 (91–97%); and chloramphenicol resistance was rare in CC258 (13%) and less frequent in CC71 (43%) compared to CC45, where it occurred in approximately 95% of the isolates (Figure 5). The differences found should reflect variations in the carriage of resistance genes among the different MRSP lineages, possibly due to truncation of the transposable elements that carry these genes, as shown recently by whole genome sequencing (McCarthy et al., 2015). Thus, our observations evidence that the therapeutic options available for MRSP infections are largely dependent on the clonal type involved. Since *tet*(M) is the most common tetracycline resistance determinant in the species, the strong association observed between CC71 and *tet*(K) confirms this to be an atypical feature of this CC, as suggested by previous studies (Perreten et al., 2010; Weese et al., 2013; Couto et al., 2014; Maaland et al., 2014).

A number of important limitations need to be considered when interpreting the data of this review. The main and unavoidable limitation is that the collection of isolates analyzed is naturally biased toward countries/continents that more often report MLST data. Therefore, some countries/continents are underrepresented in the analyzed collection (e.g., Africa, South America). Another unavoidable limitation is that we did not take into account differences in the antimicrobial susceptibility testing methods and interpretive criteria used by different studies for antimicrobial susceptibility testing. The lack of raw susceptibility data from the original studies makes it impossible to re-interpret the data according to standard interpretive criteria.

## Conclusion

MLST data analysis revealed that *S. pseudintermedius* displays an epidemic population structure and high genetic diversity, which is most likely generated by multiple recombination events rather than mutations. The five main MRSP clonal lineages that have emerged over the last 10 years are phylogenetically distinct and differ significantly with regard to SCC*mec* content, antimicrobial resistance profiles and geographical distribution. Whole genome sequencing studies are needed in the future for a better understanding of the evolution of methicillin resistance within each clonal group.

## Author contributions

LG conceived the project. TP searched the database for potentially eligible articles, extracted the data, and performed the analyses. TP, LG, PD, and AM interpreted the results. LG contacted the authors of publications when information was not provided by the articles. TP, LG, PD, and AM wrote the manuscript. All the authors reviewed the final version of the manuscript prior to submission for publication.

## Funding

The study was funded by the University of Copenhagen Research Centre for Control of Antibiotic Resistance (www.uc-care.ku.dk).

### Conflict of interest statement

The authors declare that the research was conducted in the absence of any commercial or financial relationships that could be construed as a potential conflict of interest.

## Supplementary material

Figure S1 **Phylogenetic analysis based on the nucleotide sequences of the *S. pseudintermedius* MLST genes for all 503 STs**. The analysis was based on all trees sampled with a burn-in period obtained using ClonalFrame after 200,000 iterations, including 100,000 discarded burn-ins. Scale is in coalescent units. ST71, ST68, ST45, ST112, and ST258, representing important MRSP lineages, are indicated in yellow, red, green, pink and blue, respectively. MRSP, black; MSSP, white; both MRSP and MSSP, gray.

Table S1 **Summary of MLST data from 49 studies and database on reported MRSP isolates, which accounted for 182 STs of the 503 STs defined by MLST-7**.
