# Supplementary figures and images for: Systematic Review on Global Epidemiology of Methicillin-Resistant Staphylococcus pseudintermedius: Inference of Population Structure from Multilocus Sequence Typing Data
Source: Front Microbiol. 2016 Oct 18;7:1599. doi: 10.3389/fmicb.2016.01599 (PMC5067483; doi:10.3389/fmicb.2016.01599)

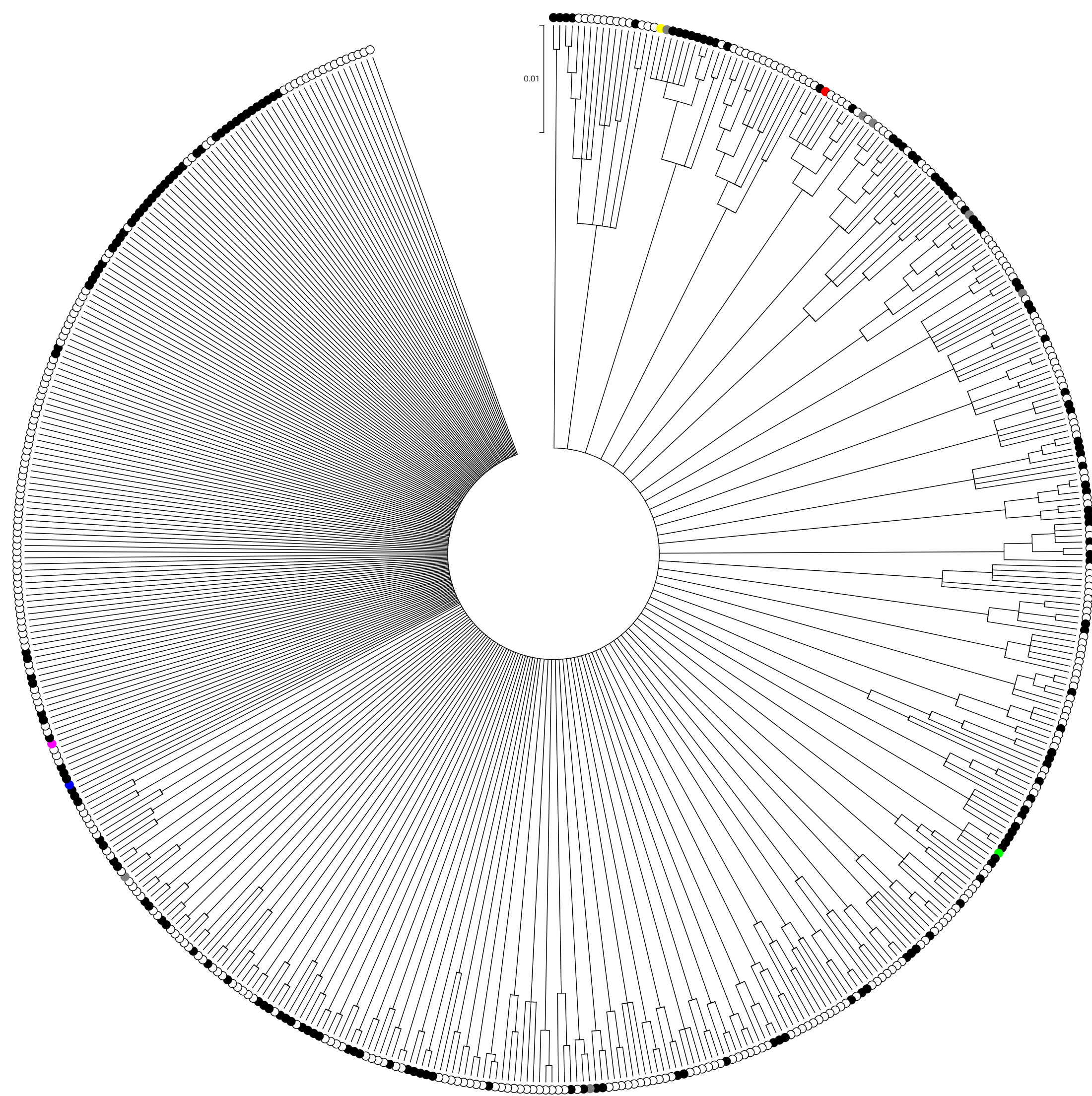

Supplement: Figure S1 — Phylogenetic analysis based on the nucleotide sequences of the S. pseudintermedius MLST genes for all 503 STs. The analysis was based on all trees sampled with a burn-in period obtained using ClonalFrame after 200,000 iterations, including 100,000 discarded burn-ins. Scale is in coalescent units. ST71, ST68, ST45, ST112, and ST258, representing important MRSP lineages, are indicated in yellow, red, green, pink and blue, respectively. MRSP, black; MSSP, white; both MRSP and MSSP, gray. [file Image1.PDF]
