# Supplementary material for: Systematic Review on Global Epidemiology of Methicillin-Resistant Staphylococcus pseudintermedius: Inference of Population Structure from Multilocus Sequence Typing Data
Source: Front Microbiol. 2016 Oct 18;7:1599. doi: 10.3389/fmicb.2016.01599 (PMC5067483; doi:10.3389/fmicb.2016.01599)
Supplement: Table S1 — Summary of MLST data from 49 studies and database on reported MRSP isolates, which accounted for 182 STs of the 503 STs defined by MLST-7. [file Table1.docx]

**Supplementary data**

**Table S1.** Summary of MLST data from 49 studies and database on reported MRSP isolates, which accounted for 182 STs of the 503 STs defined by MLST-7.

| MLST-7 | MLST-5 | CC | SCC*mec* type | *spa* type | *dru* type | Country (no. of isolates) | Origin | Host | References |
| --- | --- | --- | --- | --- | --- | --- | --- | --- | --- |
| 17 | 70 |  | V | t06 |  | USA (1) | C | human | ^1–3^ |
|  | 70 |  | V | t50 |  | South China (1) | I | dog | ^4^ |
| 29 |  | CC68 |  |  |  | the Netherlands (2) | I | dog | ^5^ |
| 41 | 69 |  | II+IV | t07 |  | Sweden (1), Norway (1) | C, unkn. | dog | ^1–3,6^ |
|  | 69 |  | NT, *ccrA2* and *ccrB2* + *ccrA4* and *ccrB4* + *mec* complex | t07 | 8u | Sweden (1), Norway (1), USA (1) | I, unkn. | dog, unkn. | ^7–10^ |
| 45 | 29, 131 | CC45 | ψSCC*mec*_57395_, NT | NT, t09 | 11a, 11ci, 11y, 11cj, 11cb, 8af, 10cm | UK (1), the Netherlands (34), Thailand (64), Israel (15), Finland (2), Denmark (3), Portugal (1) | C, I | dog, cat, human | ^1–3,11–16^ |
|  | 131 |  | NT | NT |  | the Netherlands (1) | unkn. |  | ^17^ |
| 55 | 29 | CC379 | V |  |  | Thailand (2) | I, unkn. | dog, unkn. | ^12,13^ |
|  | 29 |  | V, II-III, NT | NT, t09 |  | Japan (1), South China (4), the Netherlands (3) | C, I, unkn. | dog, cat, unkn. | ^4,17,18^ |
| 56 | 100 |  | NT |  |  | USA (2) | I | unkn. | ^2,8^ |
| 114 | 100 |  | ψSCC*mec*_57395_ |  |  | Thailand (2) | C, unkn. | dog | ^12,13^ |
| 116 | 100 | CC45 | ψSCC*mec*_57395_ |  |  | Thailand (2) | C, unkn. | dog | ^12,13^ |
|  | 100 |  | V | t23 | 11af | Norway (1), Canada (1) | I | dog, cat | ^7,10,19^ |
| 57 |  | CC45 | ψSCC*mec*_57395_ | NT |  | Israel (1) | E^*^ |  | ^13^ |
| 64 | 118 |  | III |  |  | USA (2) | I | dog, unkn. | ^2,8^ |
| 68 | 68 | CC68 | V | t06 | 11a | USA (10), Spain (4), Ireland (1), the Netherlands (1) | I, unkn. | dog, cat, horse, unkn. | ^1–3,5,9,10,20,21^ |
| 271 | 68 | CC68 | V | t06 |  | Denmark (1) | I | dog | ^16^ |
|  | 68 |  | V | t06 | 10a, 10h, 11a | USA (60), Canada (13) | I, unkn. | dog, unkn. | ^1,3,8–10,22–24^ |
| 71 | 71 | CC71 | II-III, III, NT | t02, t05, t06, t08, t23, NT |  | German (10), the Netherlands (292), Sweden (6), USA (2), Denmark (15), Switzerland (4), Italy (4), France (41), Finland (67), Japan (11), UK (2), Portugal (21), Brazil (1), Norway (12) | C, I, unkn. | dog, cat, human, unkn. | ^1–3,6,8,11,15,16,21,25–36^ |
| 169 | 71 | CC71 | II-III | t60, NT |  | USA (1), Thailand (3), Japan (11) | C, unkn. | dog, human, unkn. | ^12,13,30^ |
| 203 | 71 | CC71 | II-III | t02, t06 |  | Portugal (3) | C, I | dog | ^34^ |
| 270 | 71 | CC71 | III | t02 |  | Denmark (1) | I | dog | ^16^ |
| 272 | 71 | CC71 | III |  |  | Denmark (1) | unkn. | dog |  |
|  | 71 |  | II-III, III, IV, NT | t02, t03, t05, t06, t08, t15, NT | 8f, 9a, 9b, 10ai | Japan (18), Spain (9), Italy (17), North China (26), Norway (4), Sweden (7), the Netherlands (25), USA (13), Germany (70), Switzerland (38), Canada (20), Denmark (1) | C, I, unkn. | dog, dairy cow, unkn. | ^1,3,7–10,17–19,23,24,37–44^ |
| 84 | 26 |  | III |  |  | USA (3) | I, unkn. | dog, unkn. | ^2,8^ |
| 182 | 26 |  | V |  |  | Thailand (6) | unkn. | dog, unkn. | ^12,13^ |
|  | 26 |  | V, II, NT | NT |  | Japan (6), Norway (1), Spain (1) | C, I, unkn. | dog, unkn. | ^7,18,42^ |
| 85 |  | CC45 | ψSCC*mec*_57395_ | t09 |  | Israel (1) | I | dog | ^13^ |
| 93 | 73 |  | VII-241 |  |  | Switzerland (1) | I | dog | ^2,8,26^ |
|  | 73 |  | VII-241 | t24 | 11z | Switzerland (1) | unkn. | unkn. | ^9,10^ |
| 110 | 4 |  | V |  |  | Thailand (1) | C | human | ^12^ |
|  | 4 |  | V, NT | NT |  | South China (8) | C, I | dog, cat | ^4^ |
| 111 | 5 | CC112 | SCC*mec*_AI16_-SCC*czr*_AI16_-CI |  |  | Thailand (1) | C | dog | ^12,45^ |
| 112 | 5 | CC112 | SCC*mec*_AI16_-SCC*czr*_AI16_-CI with and without a 1,868-bp insertion; *ccrA1* and *ccrB1* + *mec* complex A | t05, t023 | 11y | Thailand (19), Germany (2) | C, I | dog, human | ^10,12,14,45^ |
|  | 5 |  | II-III, III, VII, NT | t05, t19, NT |  | South China (8), North China (8), Germany (1) | C, unkn. | dog, cat | ^4,40,44^ |
| 113 | 6 | CC45 | ψSCC*mec*_57395_ |  |  | Thailand (1) | C | human | ^12^ |
| 178 | 6 |  | V |  |  | Thailand (3) | C, unkn. | dog, human | ^12,13^ |
|  | 6 |  | V | t02, NT |  | South China (2), North China (1), | C, I, unkn. | dog | ^4,40^ |
| 115 | 133 |  | V |  |  | Thailand (1) | C | human | ^12^ |
| 181 | 133 |  | V |  |  | Thailand (14), Israel (1) | C, I, unkn. | dog, human | ^12,13^ |
|  | 133 |  | V | NT |  | South China (2) | C, I | dog, cat | ^4^ |
| 118 |  | CC258 | IV |  |  | Sweden (1), Denmark (1), the Netherlands (1) | I, unkn. | dog, human | ^5,16,46^ |
| 121 | 2 |  | V | t06 |  | Thailand (2), USA (1), Japan (1), Israel (1) | C, unkn. | dog, unkn. | ^12,13,30^ |
| 123 |  | CC71 |  |  |  | The Netherlands (2) |  | dog | ^5^ |
| 195 | 2 | CC71 | II-III | t02 |  | Portugal (1) | I | dog | ^34^ |
|  | 2 |  | II, V | t06, NT |  | Japan (1), South China (2) | I, unkn. | dog, unkn. | ^4,18^ |
| 125 | 163 |  | A |  |  | Thailand (2) | unkn. | dog, unkn. | ^12,13^ |
| 170 |  | CC68 | IV, V | t06 |  | USA (4) | C | dog, cat | ^47^ |
| 179 |  | CC45 | ψSCC*mec*_57395_ | t09 | 11a | Israel (2), the Netherlands (5) | I | dog | ^5,13^ |
| 180 |  | CC71 |  |  |  | Israel 83), Norway (1) | I, unkn. | dog, cat | ^6,13^ |
| 183 | 191 |  | V |  |  | Thailand (6) | unkn. | dog, human | ^12,13^ |
| 185 | 103 |  | ψSCC*mec*_57395_ |  |  | Thailand (4) | C, unkn. | dog, unkn. | ^12,13^ |
|  | 103 |  | V, NT | NT |  | South China (2) | C, I | dog | ^4^ |
| 193 |  | CC258 | IV | NT |  | France (1) | I | dog | ^28^ |
| 194 |  |  | II-III | t41 |  | France (1) | I | dog | ^28^ |
| 196 | 97 |  | V | t06 |  | Portugal (1), the Netherlands (2) | I | dog, cat | ^5,34^ |
| 213 | 97 |  | V | t06 |  | Portugal (1) | C | dog | ^34^ |
| 233 |  |  | V | t06 |  | South Korea (1) | C | human | ^48^ |
| 252 |  |  |  |  |  | Norway (3) | C | dog | ^6^ |
| 258 | 106 | CC258 | IV | NT |  | Denmark (2), Croatia (1), France (4), Norway (17), Italy (2), the Netherlands (49) | C, I, unkn. | dog | ^5–7,16,27,28^ |
| 273 | 106 | CC258 | IV |  |  | Denmark (1) | I | dog | ^16^ |
|  | 106 |  | IV, III | NT, t02 | 9a, 10h | Norway (7), Italy (2), the Netherlands (1), Denmark (1) | I, unkn. | dog, unkn. | ^7,9,10,41^ |
| 260 |  |  |  |  |  | UK (2) | I | dog | ^21^ |
| 261 | 111 | CC258 | IV, IVg | t05 | 10h | Denmark (1), UK (1), Croatia (1), USA (1), Norway (3), the Netherlands (31) | I, unkn. | dog, unkn. | ^5,6,9,10,16,21^ |
|  | 111 |  | NT | NT |  | the Netherlands (1) | unkn. | unkn. | ^17^ |
| 265 |  | CC258 | IV |  |  | Denmark (1), Croatia (1), the Netherlands (17) | I, unkn. | dog | ^5,16^ |
| 266 |  |  |  |  |  | Croatia (1) | I | dog |  |
| 267 | 108 | CC258 | IV | NT |  | Denmark (1) | C | dog | ^16^ |
| 268 | 86 |  | IV | t05 |  | Denmark (1) | unkn. | dog | ^16^ |
| 269 | 86 |  | IV | t05 |  | Denmark (2) | unkn. | dog | ^16^ |
| 274 |  | CC258 |  |  |  | Denmark (1) | unkn. | unkn. |  |
| 275 |  |  | III, NT | NT |  | Japan (4) | C, unkn. | dog | ^30^ |
| 276 |  |  | V | NT |  | Japan (9) |  | dog | ^30^ |
| 277 |  | CC258 | IV |  |  | Denmark (1), Italy (1), the Netherlands (2) | I | dog | ^5,16^ |
| 278 |  | CC45 |  |  |  | Italy (1) | I | dog |  |
| 279 |  | CC258 |  |  |  | Italy (1) | I | dog |  |
| 280 |  | CC258 |  |  |  | Italy (1) | I | dog |  |
| 281 |  |  |  |  |  | Israel (1) | C | dog |  |
| 282 |  | CC45 |  |  |  | Israel (1), Thailand (1), the Netherlands (1) | C, I | dog, cat | ^5,14^ |
| 283 |  |  |  |  |  | Australia (1) | unkn. | dog |  |
| 284 |  |  | V |  |  | Denmark (1) | I | dog | ^16^ |
| 285 |  |  |  |  |  | Denmark (1) | I | dog |  |
| 286 |  |  | IV |  |  | Denmark (1) | I | dog | ^16^ |
| 287 |  |  |  |  |  | Italy (1) | unkn. | dog |  |
| 288 |  | CC45 | V |  |  | Denmark (1) | I | dog | ^16^ |
| 289 |  | CC71 |  |  |  | Italy (1) | unkn. | dog |  |
| 290 |  | CC258 | IV, NT |  |  | Denmark (2) | C, unkn. | dog | ^16^ |
| 291 |  |  |  |  |  | Denmark (1) | I | dog | ^16^ |
| 293 |  |  | II-III | t06 |  | France (1) | I | dog | ^28^ |
| 294 |  |  | IV | NT |  | France (1) | I | dog | ^28^ |
| 295 |  |  | NT |  |  | Denmark (1) | I | dog | ^16^ |
| 298 |  | CC258 |  |  |  | Norway (3), the Netherlands (3) | I, unkn. | dog | ^5,6^ |
| 299 |  |  |  |  |  | Norway (3) | I, unkn. | dog | ^6^ |
| 300 |  |  |  |  |  | Norway (1) | I | dog | ^6^ |
| 301 |  | CC258 | IV |  |  | Denmark (1), Norway (1) | I, unkn. | dog | ^6^  ^16^ |
| 302 |  |  |  |  |  | Norway (1) | I | dog | ^6^ |
| 303 |  |  |  |  |  | Norway (1) | I | dog | ^6^ |
| 304 |  |  |  |  |  | Norway (1) | I | dog | ^6^ |
| 305 |  |  |  |  |  | Norway (4) | I, unkn. | dog | ^6^ |
| 306 |  | CC258 |  |  |  | Norway (1) | C | dog | ^6^ |
| 307 |  | CC258 |  |  |  | Norway (1), the Netherlands (1) | I | dog | ^5,6^ |
| 310 |  | CC45 |  |  |  | Italy (1) | I | dog |  |
| 311 |  | CC45 |  |  |  | Italy (1) | I | dog |  |
| 312 |  | CC258 |  |  |  | Italy (1), the Netherlands (2) | I | dog | ^5^ |
| 313 |  |  |  |  |  | Italy (1) | I | dog |  |
| 314 |  | CC258 |  |  |  | Italy (1) | I | dog |  |
| 315 |  | CC71 |  |  |  | Italy (1) | I | dog |  |
| 316 |  |  |  |  |  | Australia (1) | I | dog |  |
| 322 |  | CC258 |  |  |  | Spain (1) | unkn. | dog |  |
| 323 |  |  | V | t62, t58 |  | Japan (2) | I | dog | ^30^ |
| 324 |  |  | V | t02 |  | Japan (1) | I | dog | ^30^ |
| 325 |  | CC45 | IV | NT |  | Japan (1) | unkn. | dog | ^30^ |
| 330 |  |  |  |  |  | Brazil (1) | I | dog |  |
| 333 |  |  |  |  |  | the Netherlands (1) | unkn. | dog | ^5^ |
| 334 |  | CC258 |  |  |  | the Netherlands (2) | I | dog | ^5^ |
| 335 |  |  |  |  |  | the Netherlands (2) | I | dog | ^5^ |
| 336 |  | CC258 |  |  |  | the Netherlands (5) | I | dog | ^5^ |
| 337 |  |  |  |  |  | the Netherlands (1) | I | dog | ^5^ |
| 338 |  | CC68 |  |  |  | the Netherlands (1) | I | dog |  |
| 339 |  |  | NT |  |  | the Netherlands (2), Portugal (1) | I | dog | ^5,15^ |
| 340 |  | CC258 |  |  |  | the Netherlands (1) | I | dog |  |
| 341 |  |  |  |  |  | the Netherlands (1) | I | dog | ^5^ |
| 342 |  | CC258 | IV |  |  | the Netherlands (2), Portugal (2) | I | dog | ^5,15^ |
| 343 |  | CC258 |  |  |  | the Netherlands (1) | I | dog | ^5^ |
| 344 |  |  |  |  |  | the Netherlands (1) | I | dog | ^5^ |
| 345 |  |  |  |  |  | the Netherlands (1) | I | dog | ^5^ |
| 346 |  | CC258 |  |  |  | the Netherlands (3) | I | dog | ^5^ |
| 347 |  |  |  |  |  | the Netherlands (1) | I | dog | ^5^ |
| 348 |  | CC379 |  |  |  | the Netherlands (1) | I | dog | ^5^ |
| 349 |  | CC258 |  |  |  | the Netherlands (1) | I | dog | ^5^ |
| 350 |  | CC258 |  |  |  | the Netherlands (2) | I | dog | ^5^ |
| 351 |  | CC258 |  |  |  | the Netherlands (1) | I | dog | ^5^ |
| 352 |  |  |  |  |  | the Netherlands (1) | I | dog | ^5^ |
| 353 |  |  |  |  |  | the Netherlands (1) | I | dog | ^5^ |
| 354 |  | CC71 | II-III | NT |  | Japan (3) | unkn. | dog | ^30^ |
| 355 |  |  |  |  |  | Brazil (1) | I | dog |  |
| 357 |  |  |  |  |  | Sweden (1) | unkn. | dog |  |
| 358 |  | CC71 |  |  |  | Sweden (1) | unkn. | dog |  |
| 359 |  | CC112 |  |  |  | Sweden (1) | unkn. | dog |  |
| 361 |  |  |  |  |  | South Korea (1) | C | dog |  |
| 362 |  |  |  |  |  | South Korea (1) | C | human |  |
| 363 |  | CC258 |  |  |  | South Korea (1) | C | dog |  |
| 364 |  |  |  |  |  | South Korea (1) | C | dog |  |
| 365 |  | CC258 |  |  |  | South Korea (1) | C | dog |  |
| 366 |  | CC258 |  |  |  | South Korea (1) | C | dog |  |
| 367 |  |  |  |  |  | South Korea (2) | C | dog, human |  |
| 368 |  |  |  |  |  | South Korea (1) | C | dog |  |
| 369 |  | CC258 |  |  |  | Italy (2) | C, I | dog | ^27^ |
| 370 |  | CC258 |  |  |  | South Korea (1) | C | dog |  |
| 371 |  | CC112 |  |  |  | South Korea (1) | C | dog |  |
| 372 |  |  |  |  |  | South Korea (1) | C | dog |  |
| 373 |  | CC258 |  |  |  | South Korea (1) | C | dog |  |
| 379 |  | CC379 |  |  |  | South Korea (1) | C | dog |  |
| 382 |  | CC71 |  |  |  | the Netherlands (1) | unkn. | dog | ^5^ |
| 383 |  | CC258 |  |  |  | the Netherlands (1) | unkn. | dog | ^5^ |
| 384 |  |  |  |  |  | the Netherlands (1) | unkn. | dog |  |
| 388 |  |  |  |  |  | the Netherlands (1) | unkn. | dog | ^5^ |
| 389 |  | CC258 |  |  |  | the Netherlands (1) | unkn. | dog | ^5^ |
| 400 |  |  | III |  |  | Portugal (3) | I | dog | ^15^ |
| 402 |  | CC45 | NT |  |  | Finland (1) | C | dog | ^11^ |
| 403 |  |  | NT |  |  | Finland (1) | C | dog | ^11^ |
| 404 |  |  | V |  |  | Finland (1) | I | dog | ^11^ |
| 405 |  | CC258 |  |  |  | Italy (1) | I | dog |  |
| 406 |  | CC112 |  |  |  | France (1) | unkn. | dog |  |
| 407 |  |  |  |  |  | France (1) | unkn. | dog |  |
| 408 |  | CC71 |  |  |  | France (1) | unkn. | dog |  |
| 409 |  | CC45 |  |  |  | France (1) | unkn. | dog |  |
| 410 |  | CC71 |  |  |  | France (1) | unkn. | dog |  |
| 411 |  | CC71 |  |  |  | France (1) | unkn. | dog |  |
| 412 |  |  |  |  |  | France (1) | I | dog |  |
| 413 |  | CC258 |  |  |  | France (1) | unkn. | dog |  |
| 414 |  | CC258 | IV |  |  | Denmark (1), France (1) | unkn. | dog | ^16^ |
| 415 |  | CC258 |  |  |  | France (1) | unkn. | dog |  |
| 416 |  | CC258 |  |  |  | France (1) | unkn. | dog |  |
| 417 |  |  |  |  |  | France (1) | I | dog |  |
| 418 |  |  |  |  |  | France (1) | I | dog |  |
| 419 |  | CC258 |  |  |  | France (1) | unkn. | dog |  |
| 420 |  |  |  |  |  | France (1) | unkn. | dog |  |
| 421 |  |  |  |  |  | France (1) | unkn. | dog |  |
| 422 |  | CC45 |  |  |  | Israel (1) | unkn. | dog |  |
| 423 |  |  |  |  |  | Israel (1) | unkn. | dog |  |
| 426 |  |  |  |  |  | France (1) | I | dog |  |
| 427 |  |  |  |  |  | France (1) | I | dog |  |
| 428 |  |  |  |  |  | France (1) | I | dog |  |
| 429 |  | CC258 |  |  |  | Sri Lanka (1) | unkn. | dog |  |
| 430 |  | CC258 | IV |  |  | Denmark (1) | I | dog | ^16^ |
| 431 |  |  | V |  |  | Denmark (1) | unkn. | dog | ^16^ |
| 432 |  | CC112 |  |  |  | Thailand (1) | I | cat | ^14^ |
| 433 |  |  |  |  |  | Thailand (2) | I | cat | ^14^ |
| 434 |  | CC45 |  |  |  | Thailand (1) | I | cat | ^14^ |
| 461 |  |  |  |  |  | Germany (1) | I | dog |  |
| 477 |  |  |  |  |  | Italy (1) | I | dog |  |
| 496 |  |  |  |  |  | Australia (1) | I | dog |  |
| 497 |  | CC112 |  |  |  | Australia (2) | I | dog, cat |  |
| 498 |  | CC258 |  |  |  | Australia (1) | I | dog |  |
| 499 |  |  |  |  |  | Australia (1) | I | dog |  |
| 500 |  |  |  |  |  | Australia (1) | I | dog |  |
| 501 |  |  |  |  |  | Australia (1) | I | dog |  |
|  | 10 |  |  |  |  | Norway (1) | I | dog | ^7^ |
|  | 28 |  |  |  |  | Norway (2) | I | dog | ^7^ |
|  | 39 |  | V | t49 |  | South China (1) | C | dog | ^4^ |
|  | 41 |  |  |  |  | Germany (1) | C | dog, human, unkn. | ^49^ |
|  | 54 |  | NT | NT |  | South China (2) | I, unkn. | dog, unkn. | ^4,43^ |
|  | 58 |  | VII-241 | t06 | 11a | Canada (3) | unkn. | unkn. | ^9,10^ |
|  | 78 |  |  |  |  | Norway (1) | I | dog | ^7^ |
|  | 84 |  | V | t06 |  | South China (1) | I | dog | ^4^ |
|  | 92 |  | V | t06 |  | Spain (2) | C | dog, unkn | ^39,42^ |
|  | 95 |  | VII, III, V, NT | NT |  | South China (8) | I, unkn. | dog, cat, unkn. | ^4,43^ |
|  | 104 |  | V, NT | t09, NT |  | South China (6) | I | dog | ^4^ |
|  | 105 |  |  |  |  | USA (1) | I | dog | ^22^ |
|  | 112 |  | IV | t25 | 10h | USA (1) | unkn. | unkn. | ^9,10^ |
|  | 113 |  | IV | t06 | 10h | Canada (1) | unkn. | unkn. | ^9,10^ |
|  | 114 |  | III | t06 | 11v | the Netherlands (1) | unkn. | dog, unkn. | ^9,10^ |
|  | 115 |  | V, NT | NT, t21 | 9a | Japan (1), China (2), the Netherlands (1), Germany (1) | I, unkn. | dog, cat, unkn. | ^4,9,10,17,18^ |
|  | 116 |  | IV, VI | t02 | 10h | USA (1), Denmark (1) | I, unkn. | unkn. | ^8–10^ |
|  | 122 |  | III | NT |  | South China (1) | I | dog | ^4^ |
|  | 126 |  | VII, III | NT, t02 |  | South China (1), North China (2) | C, unkn. | dog, unkn. | ^4,40^ |
|  | 127 |  |  |  |  | Norway (2) | I | dog | ^7^ |
|  | 128 |  |  |  |  | Norway (1) | I | dog | ^7^ |
|  | 129 |  |  |  |  | Norway (1) | I | dog | ^7^ |
|  | 134 |  | NT, V | NT, t05 |  | South China (5) | C, I | dog, cat | ^4^ |
|  | 135 |  | VII | NT, t05, t06 |  | South China (6) | C, I | dog | ^4^ |
|  | 136 |  | III, VII | NT, t05 |  | South China (2) | C, I | dog | ^4^ |
|  | 137 |  | III | NT |  | South China (1) | I | cat | ^4^ |
|  | 138 |  | NT | NT |  | South China (1) | I | dog, unkn. | ^4^ |
|  | 139 |  | NT | NT |  | South China (1) | C | dog | ^4^ |
|  | 140 |  | III | NT |  | South China (1) | I | dog | ^4^ |
|  | 143 |  | NT | NT |  | the Netherlands (1) | unkn. | unkn. | ^17^ |
|  | 148 |  | V | NT |  | South China (1) | I | cat | ^4^ |
|  | 155 |  |  | NT |  | Thailand (1) | I | unkn. | ^14^ |

**REFERENCES**

1. Bannoehr, J., Zakour, N. L. B., Waller, A. S., Guardabassi, L., Thoday, K. L., van den Broek, A. H. M., et al. (2007). Population genetic structure of the *Staphylococcus intermedius* group: insights into *agr* diversification and the emergence of methicillin-resistant strains. *J. Bacteriol.* 189, 8685–8692. doi:10.1128/JB.01150-07.

2. Solyman, S. M., Black, C. C., Duim, B., Perreten, V., Duijkeren, E. Van, Wagenaar, J. A., et al. (2013). Multilocus sequence typing for characterization of *Staphylococcus pseudintermedius*. *J. Clin. Microbiol.* 51, 306–310. doi:10.1128/JCM.02421-12.

3. Moodley, A., Stegger, M., Ben, N. L., Fitzgerald, J. R., and Guardabassi, L. (2009). Tandem repeat sequence analysis of staphylococcal protein A (*spa*) gene in methicillin-resistant *Staphylococcus pseudintermedius*. *Vet. Microbiol.* 135, 320–326. doi:10.1016/j.vetmic.2008.09.070.

4. Feng, Y., Tian, W., Lin, D., Luo, Q., Zhou, Y., Yang, T., et al. (2012). Prevalence and characterization of methicillin-resistant *Staphylococcus pseudintermedius* in pets from South China. *Vet. Microbiol.* 160, 517–524. doi:10.1016/j.vetmic.2012.06.015.

5. Duim, B., Verstappen, K. M., Broens, E. M., Laarhoven, L. M., van Duijkeren, E., Hordijk, J., et al. (2016). Changes in the population of methicillin-resistant *Staphylococcus pseudintermedius* and dissemination of antimicrobial-resistant phenotypes in the Netherlands. *J. Clin. Microbiol.* 54, 283–288. doi:10.1128/JCM.01288-15.

6. Kjellman, E. E., Slettemeås, J. S., Small, H., and Sunde, M. (2015). Methicillin-resistant *Staphylococcus pseudintermedius* (MRSP) from healthy dogs in Norway - occurrence, genotypes and comparison to clinical MRSP. *Microbiologyopen*, 4, 857-866. doi: 10.1002/mbo3.258.

7. Osland, A. M., Vestby, L. K., Fanuelsen, H., Slettemeås, J. S., and Sunde, M. (2012). Clonal diversity and biofilm-forming ability of methicillin-resistant *Staphylococcus pseudintermedius*. *J. Antimicrob. Chemother.* 67, 841–848. doi:10.1093/jac/dkr576.

8. Black, C. C., Eberlein, L. C., Solyman, S. M., Wilkes, R. P., Hartmann, F. A., Rohrbach, B. W., et al. (2011). The role of *mecA* and *blaZ* regulatory elements in *mecA* expression by regional clones of methicillin-resistant *Staphylococcus pseudintermedius*. *Vet. Microbiol.* 151, 345–353. doi:10.1016/j.vetmic.2011.03.026.

9. Perreten, V., Kadlec, K., Schwarz, S., Grönlund Andersson, U., Finn, M., Greko, C., et al. (2010). Clonal spread of methicillin-resistant *Staphylococcus pseudintermedius* in Europe and North America: an international multicentre study. *J. Antimicrob. Chemother.* 65, 1145–1154. doi:10.1093/jac/dkq078.

10. Kadlec, K., Schwarz, S., Goering, R. V, and Weese, J. S. (2015). Direct repeat unit (*dru*) typing of methicillin-resistant *Staphylococcus pseudintermedius* from dogs and cats. *J. Clin. Microbiol.* 53, 3760–3765. doi:10.1128/JCM.01850-15.

11. Grönthal, T., Ollilainen, M., Eklund, M., Piiparinen, H., Gindonis, V., Junnila, J., et al. (2015). Epidemiology of methicillin resistant *Staphylococcus pseudintermedius* in guide dogs in Finland. *Acta Vet. Scand.* 57:37. doi:10.1186/s13028-015-0129-8.

12. Chanchaithong, P., Perreten, V., Schwendener, S., Tribuddharat, C., Chongthaleong, A., Niyomtham, W., et al. (2014). Strain typing and antimicrobial susceptibility of methicillin-resistant coagulase-positive staphylococcal species in dogs and people associated with dogs in Thailand. *J. Appl. Microbiol.* 117, 572–586. doi:10.1111/jam.12545.

13. Perreten, V., Chanchaithong, P., Prapasarakul, N., Rossano, A., Blum, S. E., Elad, D., et al. (2013). Novel pseudo-staphylococcal cassette chromosome *mec* element (ψSCC*mec*57395) in methicillin-resistant *Staphylococcus pseudintermedius* CC45. *Antimicrob. Agents Chemother.* 57, 5509–5515. doi:10.1128/AAC.00738-13.

14. Kadlec, K., Weiss, S., Wendlandt, S., Schwarz, S., and Tonpitak, W. (2016). Characterization of canine and feline methicillin-resistant *Staphylococcus pseudintermedius* (MRSP) from Thailand. *Vet. Microbiol.* 194, 93-97. doi:10.1016/j.vetmic.2016.04.015.

15. Couto, N., Monchique, C., Belas, A., Marques, C., Gama, L. T., and Pomba, C. (2016). Trends and molecular mechanisms of antimicrobial resistance in clinical staphylococci isolated from companion animals over a 16 year period. *J. Antimicrob. Chemother.* 71, 1479-1487. doi:10.1093/jac/dkw029.

16. Damborg, P., Moodley, A., Aalbæk, B., Ventrella, G., Pires dos Santos, T., and Guardabassi, L. (2016). High genotypic diversity among methicillin-resistant *Staphylococcus pseudintermedius* isolated from canine infections in Denmark. *BMC Vet. Res.* 12, 1–5. doi:10.1186/s12917-016-0756-y.

17. Laarhoven, L. M., de Heus, P., van Luijn, J., Duim, B., Wagenaar, J. A., and van Duijkeren, E. (2011). Longitudinal study on methicillin-resistant *Staphylococcus pseudintermedius* in households. *PLoS One* 6, e27788. doi:10.1371/journal.pone.0027788.

18. Bardiau, M., Yamazaki, K., Ote, I., Misawa, N., and Mainil, J. G. (2013). Characterization of methicillin-resistant *Staphylococcus pseudintermedius* isolated from dogs and cats. *Microbiol. Immunol.* 57, 496–501. doi:10.1111/1348-0421.12059.

19. Kadlec, K., Schwarz, S., Perreten, V., Andersson, U. G., Finn, M., Greko, C., et al. (2010). Molecular analysis of methicillin-resistant *Staphylococcus pseudintermedius* of feline origin from different European countries and North America. *J. Antimicrob. Chemother.* 65, 1826–1837. doi:10.1093/jac/dkq203.

20. Gómez-Sanz, E., Simón, C., Ortega, C., Gómez, P., Lozano, C., Zarazaga, M., et al. (2014). First detection of methicillin-resistant *Staphylococcus aureus* ST398 and *Staphylococcus pseudintermedius* ST68 from hospitalized equines in Spain. *Zoonoses Public Health* 61, 192–201. doi:10.1111/zph.12059.

21. McCarthy, A. J., Harrison, E. M., Stanczak-Mrozek, K., Leggett, B., Waller, A., Holmes, M. A., et al. (2015). Genomic insights into the rapid emergence and evolution of MDR in *Staphylococcus pseudintermedius*. *J. Antimicrob. Chemother.* 70, 997–1007. doi:10.1093/jac/dku496.

22. Black, C. C., Solyman, S. M., Eberlein, L. C., Bemis, D. A., Woron, A. M., and Kania, S. A. (2009). Identification of a predominant multilocus sequence type , pulsed-field gel electrophoresis cluster , and novel staphylococcal chromosomal cassette in clinical isolates of *mecA*-containing , methicillin-resistant *Staphylococcus pseudintermedius*. *Vet. Microbiol.* 139, 333–338. doi:10.1016/j.vetmic.2009.06.029.

23. DiCicco, M., Neethirajan, S., Singh, A., and Weese, J. S. (2012). Efficacy of clarithromycin on biofilm formation of methicillin-resistant *Staphylococcus pseudintermedius*. *BMC Vet. Res.* 8, 1–7. doi: 10.1186/1746-6148-8-225.

24. DiCicco, M., Weese, S., Neethirajan, S., Rousseau, J., and Singh, A. (2014). Fosfomycin susceptibility of canine methicillin-resistant *Staphylococcus pseudintermedius* isolates. *Res. Vet. Sci.* 96, 251–253. doi:10.1016/j.rvsc.2014.02.004.

25. Stegmann, R., Burnens, A., Maranta, C. A., and Perreten, V. (2010). Human infection associated with methicillin-resistant *Staphylococcus pseudintermedius* ST71. *J. Antimicrob. Chemother.* 65, 2047–2048. doi:10.1093/jac/dkq241.

26. Descloux, S., Rossano, A., and Perreten, V. (2008). Characterization of new staphylococcal cassette chromosome *mec* (SCC *mec*) and topoisomerase genes in fluoroquinolone- and methicillin-resistant *Staphylococcus pseudintermedius*. *J. Clin. Microbiol.* 46, 1818–1823. doi:10.1128/JCM.02255-07.

27. Rota, A., Corrò, M., Drigo, I., Bortolami, A., and Börjesson, S. (2015). Isolation of coagulase-positive staphylococci from bitches’ colostrum and milk and genetic typing of methicillin-resistant *Staphylococcus pseudintermedius* strains. *BMC Vet. Res.* 11, 1–7. doi:10.1186/s12917-015-0490-x.

28. Haenni, M., Alves de Moraes, N., Châtre, P., Médaille, C., Moodley, A., and Madec, J.Y. (2014). Characterisation of clinical canine meticillin-resistant and meticillin-susceptible *Staphylococcus pseudintermedius* in France. *J. Glob. Antimicrob. Resist.* 2, 119–123. doi:10.1016/j.jgar.2014.02.002.

29. Grönthal, T., Moodley, A., Nykäsenoja, S., Junnila, J., Guardabassi, L., Thomson, K., et al. (2014). Large outbreak caused by methicillin resistant *Staphylococcus pseudintermedius* ST71 in a Finnish veterinary teaching hospital – from outbreak control to outbreak prevention. *PLoS One* 9, e110084. doi:10.1371/journal.pone.0110084.

30. Ishihara, K., Koizumi, A., Saito, M., Muramatsu, Y., and Tamura, Y. (2016). Detection of methicillin-resistant *Staphylococcus pseudintermedius* ST169 and novel ST354 SCC*mec* II–III isolates related to the worldwide ST71 clone. *Epidemiol. Infect.* Jul 3, 1–9. doi:10.1017/S0950268815001545.

31. Moodley, A., Riley, M. C., Kania, S. A., and Guardabassi, L. (2013). Genome sequence of *Staphylococcus pseudintermedius* strain E140, an ST71 European-associated methicillin-resistant isolate. *Genome Announc.* 1, e00207–12. doi:10.1128/genomeA.00207-12.

32. Savini, V., Carretto, E., Polilli, E., Marrollo, R., Santarone, S., Fazii, P., et al. (2014). Small colony variant of methicillin-resistant *Staphylococcus pseudintermedius* ST71 presenting as a sticky phenotype. *J. Clin. Microbiol.* 52, 1225–1227. doi:10.1128/JCM.02861-13.

33. Starlander, G., Börjesson, S., Grönlund-Andersson, U., Tellgren-Roth, C., and Melhus, Å. (2014). Cluster of infections caused by methicillin-resistant *Staphylococcus pseudintermedius* in humans in a tertiary hospital. *J. Clin. Microbiol.* 52, 3118–3120. doi:10.1128/JCM.00703-14.

34. Couto, N., Belas, A., Couto, I., Perreten, V., and Pomba, C. (2014). Genetic relatedness, antimicrobial and biocide susceptibility comparative analysis of methicillin-resistant and -susceptible *Staphylococcus pseudintermedius* from Portugal. *Microb. Drug Resist.* 20, 364–371. doi:10.1089/mdr.2013.0043.

35. Haenni, M., Châtre, P., Keck, N., Franco, A., Battisti, A., and Madec, J.Y. (2013). Hospital-associated meticillin-resistant *Staphylococcus pseudintermedius* in a French veterinary hospital. *J. Glob. Antimicrob. Resist.* 1, 225–227. doi:10.1016/j.jgar.2013.05.005.

36. Quitoco, I. M. Z., Ramundo, M. S., Silva-Carvalho, M. C., Souza, R. R., Beltrame, C. O., de Oliveira, T. F., et al. (2013). First report in South America of companion animal colonization by the USA1100 clone of community-acquired meticillin-resistant *Staphylococcus aureus* (ST30) and by the European clone of methicillin-resistant *Staphylococcus pseudintermedius* (ST71). *BMC Res. Notes* 6, 1–7. doi:10.1186/1756-0500-6-336.

37. Gómez-Sanz, E., Torres, C., Ceballos, S., Lozano, C., and Zarazaga, M. (2013b). Clonal dynamics of nasal *Staphylococcus aureus* and *Staphylococcus pseudintermedius* in dog-owning household members. Detection of MSSA ST398. *PLoS One* 8, e69337. doi:10.1371/journal.pone.0069337.

38. Pilla, R., Bonura, C., Malvisi, M., Snel, G. G. M., and Piccinini, R. (2013). Methicillin-resistant *Staphylococcus pseudintermedius* as causative agent of dairy cow mastitis. *Vet. Rec.* 173:19. doi:10.1136/vr.101485.

39. Gómez-Sanz, E., Torres, C., Lozano, C., and Zarazaga, M. (2013c). High diversity of *Staphylococcus aureus* and *Staphylococcus pseudintermedius* lineages and toxigenic traits in healthy pet-owning household members. Underestimating normal household contact? *Comp. Immunol. Microbiol. Infect. Dis.* 36, 83–94. doi:10.1016/j.cimid.2012.10.001.

40. Wang, Y., Yang, J., Logue, C. M., Liu, K., Cao, X., Zhang, W., et al. (2012). Methicillin-resistant *Staphylococcus pseudintermedius* isolated from canine pyoderma in North China. *J. Appl. Microbiol.* 112, 623–630. doi:10.1111/j.1365-2672.2012.05233.x.

41. Paul, N. C., Moodley, A., Ghibaudo, G., and Guardabassi, L. (2011). Carriage of methicillin-resistant *Staphylococcus pseudintermedius* in small animal veterinarians: indirect evidence of zoonotic transmission. *Zoonoses Public Health* 58, 533–539. doi:10.1111/j.1863-2378.2011.01398.x.

42. Gómez-Sanz, E., Torres, C., Lozano, C., Sáenz, Y., and Zarazaga, M. (2011). Detection and characterization of methicillin-resistant *Staphylococcus pseudintermedius* in healthy dogs in La Rioja, Spain. *Comp. Immunol. Microbiol. Infect. Dis.* 34, 447–453. doi:10.1016/j.cimid.2011.08.002.

43. Boost, M. V, So, S. Y. C., and Perreten, V. (2011). Low rate of methicillin-resistant coagulase-positive staphylococcal colonization of veterinary personnel in Hong Kong. *Zoonoses Public Health* 58, 36–40. doi:10.1111/j.1863-2378.2009.01286.x.

44. Ruscher, C., Lübke-Becker, Semmler, T., Wleklinski, C.G., Paasch, A., Soba, A., et al. (2010). Widespread rapid emergence of a distinct methicillin- and multidrug-resistant *Staphylococcus pseudintermedius* (MRSP) genetic lineage in Europe. *Vet. Microbiol.* 144, 340–346. doi:10.1016/j.vetmic.2010.01.008.

45. Chanchaithong, P., Prapasarakul, N., Perreten, V., and Schwendener, S. (2015). Characterization of a novel composite staphylococcal cassette chromosome *mec* in methicillin-resistant *Staphylococcus pseudintermedius* from Thailand. *Antimicrob. Agents Chemother.* 60, 1153–1157. doi:10.1128/AAC.02268-15.

46. Börjesson, S., Gómez-Sanz, E., Ekström, K., Torres, C., and Grönlund, U. (2015). *Staphylococcus pseudintermedius* can be misdiagnosed as *Staphylococcus aureus* in humans with dog bite wounds. *Eur. J. Clin. Microbiol. Infect. Dis.* 34, 839–844. doi:10.1007/s10096-014-2300-y.

47. Davis, J. A., Jackson, C. R., Fedorka-Cray, P.J., Barrett, J. B., Brousse, J. H., Gustafson, J., et al. (2014). Carriage of methicillin-resistant staphylococci by healthy companion animals in the US. *Lett. Appl. Microbiol.* 59, 1–8. doi:10.1111/lam.12254.

48. Youn, J.H., Moodley, A., Park, Y.H., and Sugimoto, C. (2013). Genome sequence of methicillin-resistant *Staphylococcus pseudintermedius* sequence type 233 (ST233) strain K7, of human origin. *Genome Announc.* 1, e00310–13. doi:10.1128/genomeA.00310-13.

49. Walther, B., Hermes, J., Cuny, C., Wieler, L. H., Vincze, S., Abou, Y., et al. (2012). Sharing more than friendship — nasal colonization with coagulase-positive staphylococci (CPS) and co-habitation aspects of dogs and their owners. *PLoS One* 7, e35197. doi:10.1371/journal.pone.0035197.
